# Association Between Occupational Heat Stress and Kidney Disease Among 37 816 Workers in the Thai Cohort Study (TCS)

**DOI:** 10.2188/jea.JE20110082

**Published:** 2012-05-05

**Authors:** Benjawan Tawatsupa, Lynette L-Y Lim, Tord Kjellstrom, Sam-ang Seubsman, Adrian Sleigh, the Thai Cohort Study Team

**Affiliations:** 1 Health Impact Assessment Division, Department of Health, Ministry of Public Health, Nonthaburi, Thailand; 2 National Centre for Epidemiology and Population Health, The Australian National University, Canberra ACT, Australia; 3 Centre for Global Health Research, Umeå University, Umeå, Sweden; 4 Thai Health-Risk Transition: National Cohort Study, School of Human Ecology, Sukhothai Thammathirat Open University, Nonthaburi, Thailand

**Keywords:** occupational heat stress, kidney disease, Thai Cohort Study, Thailand

## Abstract

**Background:**

We examined the relationship between self-reported occupational heat stress and incidence of self-reported doctor-diagnosed kidney disease in Thai workers.

**Methods:**

Data were derived from baseline (2005) and follow-up (2009) self-report questionnaires from a large national Thai Cohort Study (TCS). Analysis was restricted to full-time workers (*n* = 17 402 men and 20 414 women) without known kidney disease at baseline. We used logistic regression models to examine the association of incident kidney disease with heat stress at work, after adjustment for smoking, alcohol drinking, body mass index, and a large number of socioeconomic and demographic characteristics.

**Results:**

Exposure to heat stress was more common in men than in women (22% vs 15%). A significant association between heat stress and incident kidney disease was observed in men (adjusted odds ratio [OR] = 1.48, 95% CI: 1.01–2.16). The risk of kidney disease was higher among workers reporting workplace heat stress in both 2005 and 2009. Among men exposed to prolonged heat stress, the odds of developing kidney disease was 2.22 times that of men without such exposure (95% CI 1.48–3.35, *P*-trend <0.001). The incidence of kidney disease was even higher among men aged 35 years or older in a physical job: 2.2% exposed to prolonged heat stress developed kidney disease compared with 0.4% with no heat exposure (adjusted OR = 5.30, 95% CI 1.17–24.13).

**Conclusions:**

There is an association between self-reported occupational heat stress and self-reported doctor-diagnosed kidney disease in Thailand. The results indicate a need for occupational health interventions for heat stress among workers in tropical climates.

## INTRODUCTION

The rising frequency of very hot days and the spread of urban heat-island effects in many cities are affecting the health of elderly populations.^1^ Working people are also affected by heat stress, especially outdoor workers exposed to excessive heat because of their job.^1^ Health impacts from elevated air temperature have been observed in many studies, and respiratory, cardiovascular, and kidney disease have all been linked to global warming.^1^^–^^4^ Moreover, occupational heat stress was found to be associated with worse mental health and psychological distress in Thailand.^5^

The body’s natural methods of cooling include convection, conduction, and evaporation of sweat. Only evaporation will lower body temperature when air temperature is higher than 35°C (which is common in tropical countries), and it is less effective when humidity is high.^6^ Evaporation leads to loss of body water and electrolytes, especially sodium and chloride, which are responsible for maintenance of overall fluid balance. Depletion of water and sodium results in loss of extracellular fluid volume, which can place acute or chronic stress on kidney function and ultimately lead to kidney disease.^7^

Kidney disease is usually divided into 2 forms: acute kidney failure (sudden loss of kidney function) and chronic kidney failure (slow, gradual loss of kidney function). Acute kidney failure can result from severe dehydration. Slow loss of kidney function is exacerbated by diabetes, hypertension, and blockage from kidney stones. Kidney stones are more common with chronic dehydration.^8^ Heat waves and related dehydration are associated with both acute renal failure and chronic renal disease.^2^^,^^4^^,^^9^^–^^11^

In Thailand, kidney disease is a major cause of death among middle-aged adults. The number of deaths from renal failure has increased from 8895 in 2001 to 11 246 in 2005 and 12 195 in 2007.^12^ A high incidence of urolithiasis (kidney stones) with a male/female ratio of 1.6 to 2 was observed among manual workers (farmers, laborers, housekeepers), especially farmers.^13^^,^^14^ The increase in renal deaths and high incidence of urolithiasis in manual workers are worrisome because they might be partially caused by increasing heat stress in a hot, humid country with dynamic economic development. From 1951 to 2003, the mean maximum daily temperature in Thailand increased by 0.56°C, and the mean minimum temperature increased even more, by 1.44°C.^15^ Increasing heat stress is anticipated as Thailand urbanizes, due to the urban heat-island effect and the continuing increase in average temperature associated with global warming.

A related issue is the effect of occupational heat stress, especially in tropical countries, where temperatures and other climate variables are already thermally stressful.^16^ Problems regarding heat exposure in occupational settings exist in Thailand,^17^ but there are no studies of heat-related kidney disease. This study examines the association between occupational exposure to heat stress and kidney disease. The data are derived from the self-reports of a large national cohort of Thai distance learning students of Open University whose social and physical environment and health outcomes have been followed from 2005 to 2009.^18^

## METHODS

The Thai Cohort Study (TCS) is an ongoing examination of the health-risk transition in the adult Thai population. At baseline in 2005, all cohort members were enrolled as distance learning students at Sukhothai Thammathirat Open University (STOU), resided all over Thailand, lived with their families, and worked part- or full-time. The study started with a 2005 mailed baseline questionnaire that inquired about sociodemographic variables, work, health and injuries, social networks and well-being, diet and physical activity, tobacco, alcohol, and transport. A follow-up questionnaire in 2009 repeated some of the sociodemographic and occupational measurements and obtained information on heat stress and other work hazards, well being, mental health, self-rated health, and information on specific diseases, including kidney disease. There were 87 134 respondents at baseline, and 70% (60 569) responded to the follow-up study. Those who were included in the 2009 follow-up were the same persons as those studied in 2005, and the 2 data sets have been linked.^18^^–^^20^

Occupational heat stress in 2005 was assessed by the question: “During the last 12 months, how often did you experience high temperatures that made you uncomfortable at work? (in 2005)”, to which respondents answered on a 4-point scale: often, sometimes, rarely, and never. For analysis of 2005 heat stress, the scale was collapsed to 3 categories by combining rarely and never. Heat stress in 2009 was evaluated using the question: “How often did the hot period this year interfere with your work? (in 2009)”, to which respondents answered on a 5-point scale: every day, 1 to 6 times/wk, 1 to 3 times/month, never, and not applicable (N/A; use air conditioning). These 2009 data were combined with 2005 data to create a 2005–2009 heat stress variable as follows: never heat stress (never or rarely in 2005 and N/A or ≤1–3 times/month in 2009); non-prolonged heat stress (sometimes or often in 2005 and N/A or ≤1–3 times/month in 2009, or vice versa, ie, rarely or never in 2005 and ≥1–6 times/wk in 2009); prolonged heat stress (sometimes or often in 2005 and ≥1–6 times/wk in 2009).

The health outcomes analyzed here were based on self-reports in 2005 and 2009 in response to the question “Have you ever been diagnosed by a doctor as having kidney disease?”, with answers of yes and no. We identified incident cases as patients who did not report a diagnosis of kidney disease at baseline in 2005 but did report such a diagnosis in 2009. Also, those who answered yes in 2009 were requested to indicate their age at diagnosis. This enabled an alternative (more restrictive) method to identify incident cases by using reported age at diagnosis and calculated age in 2009 to impute onset of kidney disease after the 2005 baseline. We used the first method of identifying incident cases for our main analyses; the alternative method was used to check our primary results.

**Figure 1.**
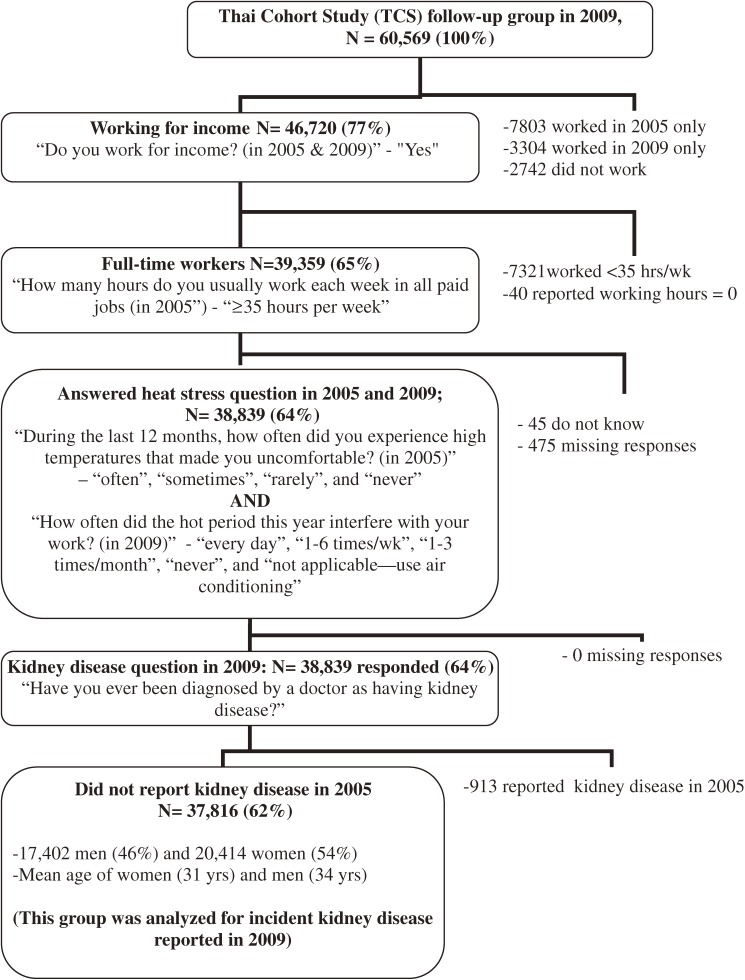
Selection process for analyzed population from the Thai Cohort Study, using data from 2005 and 2009

The process used to select the cohort members who were analyzed for incidence of kidney disease is shown in Figure 1. The respondents included were those who did not report kidney disease at cohort baseline in 2005, had a paid job in both 2005 and 2009, and answered the questions on heat exposure in 2005 (for analysis of baseline exposure) and both 2005 and 2009 (for analysis of prolonged exposure). They also provided information in 2005 on variables that could confound or modify our estimates of the association between heat stress and kidney disease. These variables were age, sex, education, income, alcohol consumption, smoking, job location, and job type. Because we restricted our study of heat stress to a longitudinal analysis of workers, we did not include individuals who worked in 2005 but did not work in 2009 (Figure 1). In 2009, these 7803 excluded persons reported incident kidney disease at a rate almost identical (1.19%) to that noted in the analyzed cohort of 37 816 persons (1.07%). Thus, there is no evidence that their exclusion lowered our estimate of incident kidney disease.

To prepare for the analysis, we divided heat stress, age, highest education, monthly income, alcohol consumption, smoking, job location, and job type into appropriate categories, and frequencies were tabulated by sex (Table 1). Classification of employment as an office job or physical job was based on the more detailed Thai categories that were reported: skilled and manual workers were coded as physical jobs; all others were coded as office jobs. Body mass index (BMI) was derived from reported height and weight at endpoint (2009) and was classified into 4 categories (underweight, normal weight, overweight, and obese).

**Table 1.** Reported heat stress at work and socioeconomic, behavioral, and other characteristics in a cohort of 37 816 full-time workers in Thailand

Cohort attributes	Men		Women	
	
	*n*	%	*n*	%
Total	17 402	46.0	20 414	54.0
Heat stress in 2005				
Rarely/Never	7523	43.2	11 288	55.3
Sometimes	5992	34.4	6008	29.4
Often	3887	22.3	3118	15.3
Age group (years)				
15–24	1989	11.4	4411	21.6
25–34	8043	46.2	10 196	50.0
≥35	7370	42.4	5807	28.5
Education				
University	4499	25.9	6472	31.7
Diploma	4422	25.4	6885	33.7
High school	8481	48.7	7057	34.6
Personal income (Baht/month)^a^				
20 001+	2925	16.8	2180	10.7
10 001–20 000	6162	35.4	5573	27.3
7001–10 000	4385	25.2	5450	26.7
<7000	3930	22.6	7211	35.3
Alcohol consumption				
Never	1494	8.6	8033	39.4
Occasional social drinker	12 387	71.2	10 882	53.3
Regular drinker	1971	11.3	124	0.6
Stopped drinking	1550	8.9	1375	6.7
Smoking				
Never smoked	8337	47.9	19 531	95.7
Current smoker	3424	19.7	172	0.8
Ex-smoker	5641	32.4	711	3.5
BMI in 2009				
Normal weight	6966	40.0	11 609	56.9
Underweight	538	3.1	2707	13.3
Overweight	4500	25.9	2844	13.9
Obese	5398	31.0	3254	15.9
Job location				
Bangkok	2576	14.8	3965	19.4
Urban	6712	38.6	7418	36.3
Rural	8114	46.6	9031	44.2
Job type				
Office job	12 393	71.2	16 569	81.2
Physical job	5009	28.8	3845	18.8

^a^US$ 1 = 40 Baht in 2005.

### Data analysis

Data were digitized using Thai Scandevet software and further processed using SPSS version 19 and Stata version 12 statistical packages. The initial analysis group included 37 816 cohort members who worked full-time from 2005 through 2009 and full data sets for heat stress and kidney disease analyses. Subsequent analyses were restricted to 17 402 men who had a significant risk of heat stress and related kidney disease.

**Figure 2.**
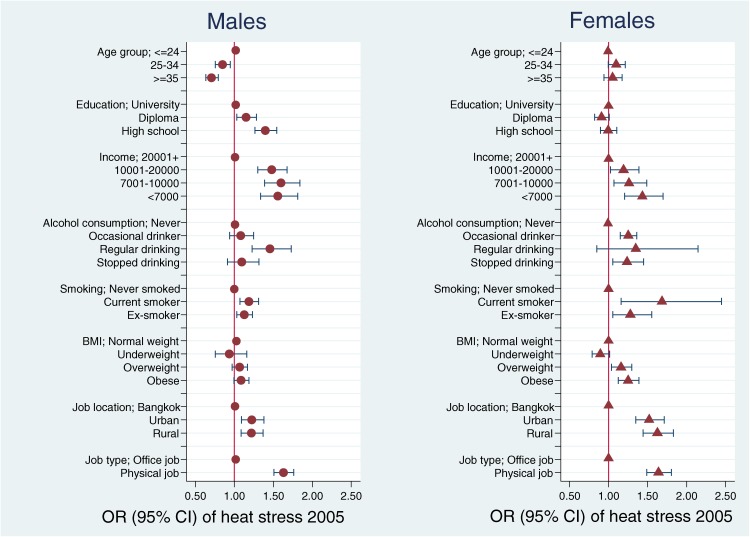
Association of cohort attributes with heat stress in 2005^a^  ^a^Odds ratios (ORs) for age not adjusted; other ORs mutually adjusted and adjusted for age

**Figure 3.**
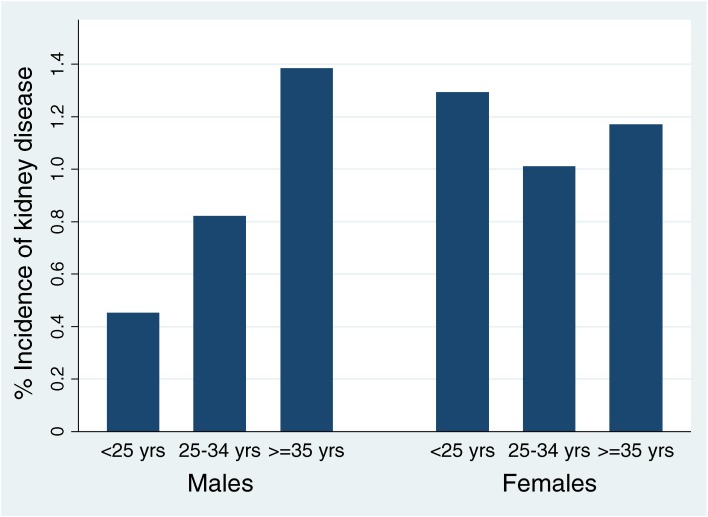
Incidence of kidney disease by age group among male and female workers, 2005–2009

Potential explanatory variables were first assessed by investigating their association (odds ratios [ORs] and 95% CIs) with the exposure of interest (heat stress in 2005), with results shown graphically for men and women (Figure 2). For this summary analysis, heat stress in 2005 was dichotomized into often and not often (sometimes/rarely/never). The cumulative incidence of kidney disease was then calculated for the period 2005–2009 by age group and sex (Figure 3). Age trends were tested for statistical significance (*P*-trend <0.05).

For the main analyses, we use logistic regression models with incident kidney disease as the outcome to estimate the association with exposure to heat stress, and calculated crude and adjusted ORs and 95% CIs. Adjusted estimates were derived from models that included all potential explanatory variables. These crude and adjusted models were then subjected to a sensitivity analysis by repeating the estimations using an alternative, more restrictive method of defining incident kidney disease (based on a negative report of kidney disease in 2005, a positive report in 2009, and age at diagnosis of kidney disease reported in 2009, after confirming kidney disease-free status in 2005).

Putative explanatory variables were also evaluated as potential modifiers of heat stress associations by stratified analyses that estimated ORs separately for each variable category. The significant modifiers of heat stress effects on kidney disease were age and job type in men. The final models show the fully adjusted heat stress effect on incidence of kidney disease, stratified by age and job type in male workers.

### Ethics

Ethical approval was obtained from Sukhothai Thammathirat Open University Research and Development Institute (protocol 0522/10) and the Australian National University Human Research Ethics Committee (protocol 2004344). Informed, written consent was obtained from all participants.

## RESULTS

### Characteristics of the cohort

The selection of the analyzed cohort of 2005–2009 workers is presented in Figure 1. Table 1 shows the main characteristics of the 17 402 men (46.0%) and 20 414 women (54.0%) analyzed. The most frequent age range was 25 to 34 years. Women were younger and more likely to work in Bangkok than men (19.4% vs 14.8%). Men were more likely than women to have physical jobs (28.8% vs 18.8%). On average, women had a moderately higher educational level, while men had higher personal incomes. Overall, many more men than women were current smokers (19.7% vs 0.8%) and regular drinkers (11.3% vs 0.6%). More men than women were obese (31.0% vs 15.9%). Exposure to heat stress at baseline (2005) was more common in men than in women (22.3% vs 15.3%). Never experiencing heat stress at work was more frequently reported by women than by men (55.3% vs 43.2%). Further analyses were therefore conducted separately for men and women.

### Associations of cohort attributes with heat stress

The associations of cohort attributes with heat stress among men and women in 2005 are presented in Figure 2. The age association with heat stress is shown directly (ie, unadjusted for covariates); other variables tested are mutually adjusted for their association with heat stress. Among both sexes, the prevalence of heat stress exposure varied considerably among subgroups and was notably higher for those with lower incomes and education, regular drinkers, and current smokers. Workers in rural areas more frequently reported heat stress at work than did workers in Bangkok. Those with physical jobs had more heat stress at work than did office workers. For men, all explanatory variables except BMI were significantly associated with heat stress. For women, all explanatory variables except age and education were significantly associated with heat stress. Among workers aged 35 years or older, reporting both heat stress and a physical job was much more common among men (509) than among women (181).

### Incidence of kidney disease

The incidence of kidney disease by age group and sex during the 4-year period from 2005 to 2009 is presented in Figure 3. Overall, 405 (1.1%) of the analyzed group developed kidney disease, and the rate was very similar for men and women (1.0% vs 1.1%). Incident kidney disease increased significantly with age for men, reaching 1.4% among those aged 35 years or older (*P*-trend <0.001); in contrast, there was no age trend for women (*P*-trend = 0.664). These results show that age is a potential confounder of heat stress effects for men because the proportion of heat stress exposure is greater among younger men, while the incidence of kidney disease increases with age.

### Effect of occupational heat stress on kidney disease

Table 2 shows crude and adjusted ORs for associations between heat stress and incident kidney disease for men and women. For men, the incidence of kidney disease from 2005 to 2009 was 1.3% among those exposed to heat stress in 2005, compared with 0.9% among those not exposed (adjusted OR = 1.48, 95% CI 1.01–2.16). For men, there was a significant dose-response relation between heat stress and kidney disease (*P*-trend for adjusted OR = 0.046). There was no similar trend for women. Therefore, subsequent analyses of the effect of heat stress on kidney disease in different age groups and job types were restricted to men.

**Table 2.** Associations between heat stress and incident kidney disease among men and women working full-time

Heat stress 2005	Kidney disease		ORs			*P*-value	95% CI
	
	No.	%	Crude	Age adj.^a^	Adj.^b^		
Men (*n* = 17 402)	177	1.02					
Never/Rarely	66	0.88	1	1	1		
Sometimes	62	1.03	1.18	1.21	1.19	0.345	0.83–1.69
Often	49	1.26	1.44	1.54*	1.48*	0.045	1.01–2.16
*P*-trend			0.054	0.025	0.046		

Women (*n* = 20 414)	228	1.12					
Never/Rarely	130	1.15	1	1	1		
Sometimes	65	1.08	0.94	0.94	0.91	0.548	0.67–1.23
Often	33	1.06	0.92	0.92	0.87	0.471	0.59–1.28
*P*-trend			0.604	0.606	0.411		

(*P-value <0.05) & (**P-value <0.001).

^a^Associations with heat stress and incidence of kidney disease are expressed as age-adjusted odds ratios (ORs).

^b^Associations with heat stress and incidence of kidney disease are expressed as ORs adjusted for explanatory variables: age, alcohol consumption, smoking, body mass index, income, education, job type, and job location.

**Figure 4.**
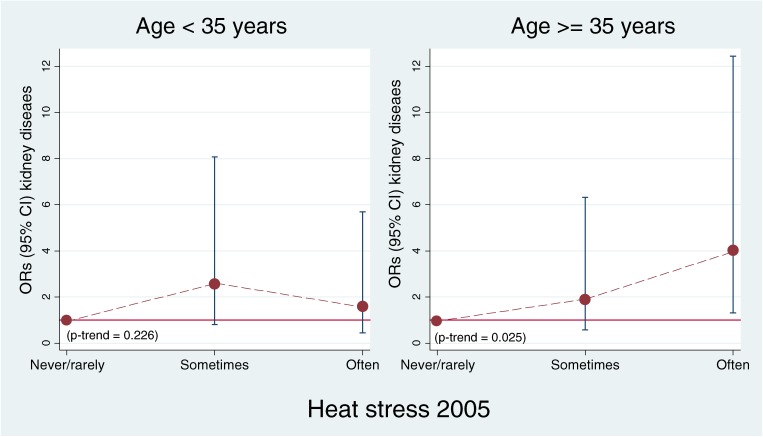
Effect of heat stress in 2005 on incidence of kidney disease in 2009 among men in physical jobs

Compared with men not exposed to heat stress, men with physical jobs who were exposed to heat stress were at higher risk of kidney disease (adjusted OR: 2.57, 95% CI: 1.11–5.93) than were heat-stressed office workers (adjusted OR: 1.26, 95% CI: 0.80–1.98) (data not tabulated). In particular, men aged 35 years or older in physical jobs with frequent heat stress had a particularly high risk of kidney disease (2.6%) compared with the same age group in physical jobs without heat stress (0.7%). In a comparison of these 2 groups, the adjusted OR for kidney disease associated with frequent heat stress was 4.33 (95% CI 1.38–13.60). Moreover, the odds increased with increasing heat stress exposure in this age group (*P*-trend = 0.025; Figure 4).

### Effect of prolonged heat stress on kidney disease

**Figure 5.**
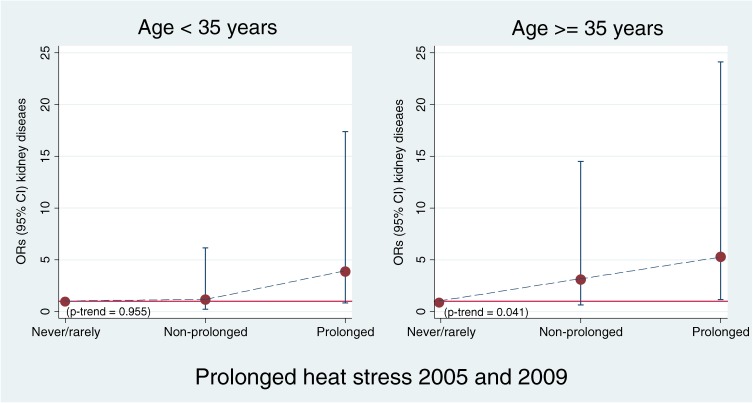
Effect of prolonged heat stress (2005 and 2009) on incidence of kidney disease among men in physical jobs

Overall, 26.1% of male workers were exposed to prolonged heat stress in both 2005 and 2009. Working under prolonged heat stress was notably more frequent among those who were younger, had lower incomes and education, regular drinkers, current smokers, those who worked in rural areas, and particularly those with physical jobs (data not shown); this is the same pattern that was noted above for heat stress at baseline (2005). With increasing exposure to prolonged heat stress among men, the adjusted OR of developing kidney disease also increased (*P*-trend <0.001). Among those frequently exposed to heat stress, the adjusted OR reached 2.22 (95% CI 1.48–3.35; Table 3). The risk of kidney disease was highest for men aged 35 years or older with frequent prolonged heat stress in physical jobs: 2.2% of such men developed kidney disease compared with 0.4% among those without heat stress (adjusted OR = 5.30, 95% CI 1.17–24.13). In addition, men in this age group with physical jobs had a significantly increased risk of kidney disease with increasing prolonged heat exposure (*P*-trend = 0.041; Figure 5).

**Table 3.** Associations between prolonged heat stress and incident kidney disease among men working full-time

Prolonged heat stress (2005 and 2009)	Men %	Kidney disease		ORs			*P*-value	95% CI
	
		No.	%	Crude	Age adj.^a^	Adj.^b^		
Men (*n* = 17 402)		177	1.02					
Never	31.5	39	0.71	1	1	1		
Non-prolonged heat	42.5	71	0.96	1.35	1.42	1.40	0.099	0.94–2.08
Prolonged heat stress ​ 2005 and 2009	26.0	67	1.48	2.09**	2.25**	2.22**	<0.001	1.48–3.35
*P*-trend				<0.001	<0.001	<0.001		

(*P-value <0.05) & (**P-value <0.001).

^a^Associations with heat stress and incidence of kidney disease are expressed as age-adjusted odds ratios (ORs).

^b^Associations with heat stress and incidence of kidney disease are expressed as ORs adjusted for explanatory variables: age, alcohol consumption, smoking, BMI, income, education, job type and job location.

### Sensitivity analysis using an alternative method of defining incident kidney disease

The above analyses were repeated using the alternative method of estimating the incidence of kidney disease, ie, restricted to those who were negative for kidney disease in 2005, positive in 2009, and, in 2009, reported age at diagnosis (indicating disease onset) after 2005. This method yielded 30% fewer cases of incident kidney disease among full-time workers (283 vs 405), which reduced statistical power. Crude and adjusted ORs for incident kidney disease associated with heat stress in 2005 among men and women were almost identical to corresponding ORs reported in Table 2, ie, using the primary method of estimating incidence. In addition, using the 2005–2009 heat stress variable, the effect of prolonged heat stress among men was identical to the corresponding OR in Table 3 (adjusted OR: 2.22), and the overall effects of heat stress (never vs non-prolonged vs prolonged) remained highly significant for trend (<0.001).

## DISCUSSION

Several findings in the present study of occupational heat stress and kidney disease were notable. Many Thai workers (18%) in the cohort reported exposure to heat stress at baseline in 2005, and such exposure was more common in men than in women. Furthermore, there was an association between reported heat stress and 4-year (2005–2009) incidence of kidney disease in men but not in women. This important finding was validated by showing almost identical results when defining incidence using an alternative method based on reported age at diagnosis. These results were tested yet again by including only kidney disease cases with an institution named for diagnosis, and again the results were similar (data not shown). We also found that the risk of kidney disease increased with increasing dose of prolonged heat stress exposure in men and that the risk was higher among those aged 35 years or older and those with physical jobs. Older men in physical jobs had a 5.3-times increase in the risk of incident kidney disease.

Overall, these epidemiologic results remained substantial and statistically significant when adjusted for a variety of potential explanatory variables (age, income, education, alcohol consumption, smoking, BMI, job type, and job location). These included adjustments for socioeconomic status—which has been shown in other studies to be an important correlate of kidney disease^21^^,^^22^—and for smoking.^23^

The main advantage of this study is its access to a large nationwide cohort of 17 402 male and 20 414 female full-time workers in Thailand. It reasonably accurately represents male and female Thais of working age with regard to geographic location, age, and socioeconomic status.^18^^–^^20^ Cohort members are better educated than average Thais of the same age and sex, which enabled us to gather complex heat exposure and health outcome data by questionnaire. The cohort showed a wide range of values for the variables of interest, which allowed us to investigate the relationship between heat stress at work and health outcomes. Furthermore, the follow-up study allows us to analyze incidence data and to categorize heat exposure by degree of prolongation.

Another strength of this study is that self-reports of heat stress were unlikely to be biased, as the questionnaires included a substantial number of questions on different exposures and diseases, and thus respondents would be unaware of any connection to kidney disease. Also, they would not benefit by over- or under-reporting.

We found an association of reported heat stress at work and reported kidney disease in tropical Thailand. However, this important overall finding has some limitations. Our study could not directly establish that kidney disease resulted from heat stress. However, the longitudinal data allow us to be sure that heat stress exposure preceded kidney disease outcomes. Unfortunately, the source and nature of heat stress, and actual work conditions, were not characterized in detail. We were not able to directly measure, or further investigate, work environments or kidney disease outcomes and hence this report must be regarded as preliminary. Therefore, there is a need for more detailed observations in informative, heat-stressed work settings and for studies of actual kidney disease to validate our findings, explore underlying mechanisms, and further characterize associated kidney disease.

As noted above, we need to collect more detailed health and environmental information from various work settings in Thailand. We should begin with an informative sample of the cohort analyzed in this report. The sample should include both sexes, a variety of occupations, and participants aged 35 years or older in urban and rural settings of major regions of Thailand. The sample must also include persons with or without heat stress and with or without kidney disease. We could collect detailed information on heat stress, water intake and dehydration, and reported kidney disease. Wet-bulb temperature measurements at workplaces of a subsample of affected individuals would permit us to document heat stress, and medical records could document kidney disease and its characteristics. Such a study should lead to more detailed assessment of other (non-cohort) samples of at-risk heat-stressed workers and would include extensive clinical examinations of the kidney to conclusively document renal effects.

Our findings complement those of other reports that highlight the growing importance of kidney disease in Thailand. The Thai Ministry of Public Health reports a 70% increase in hospitalizations for kidney disease (acute renal failure, chronic renal failure, and kidney stone) from 2005 (180 779 cases) to 2009 (305 130 cases).^24^ The Fourth National Health Examination Survey Report 2008–2009 found that the prevalence of renal failure was 1.2% among Thai adults, and the rates were similar for men and women.^25^ In our cohort, the incidence of reported doctor-diagnosed kidney disease was 1.1%, with similar rates for men and women. However, kidney disease among women did not appear to involve heat stress and was probably related to female anatomy—short urethras increase the incidence of urinary tract infection. However, among men, occupational heat stress was a risk factor for kidney disease. This may be because, compared with women, the proportions of men who work in physical jobs and work outdoors are higher.^26^ Women have more body fat and are consequently more sensitive to heat. Men could also be less aware of dehydration and engage in more-intense muscular activity, resulting in hyperuricemia and rhabdomyolysis, both of which pose a threat to kidney health.^27^ It should also be noted that men appear to be more susceptible to heat stress, as shown by data from a 2003 heat wave in France.^28^

Although there is a high prevalence of kidney disease among outdoor workers in the tropics, occupational heat stress-related kidney disease has received little attention.^29^ In an Italian study, Borghi et al^30^ showed that chronic dehydration among glass-industry workers exposed to heat stress was a risk factor for kidney stones. In a Brazilian study, Atan et al^31^ found that male steel-industry workers in high-temperature areas had a 9-fold risk of kidney stones compared with those working at room temperature. A study by Gracia-Trabanino et al^32^ found a high prevalence of chronic kidney disease among coastal male farmers in Central American countries. Recently, other reports have shown an association between extreme temperature (heat waves) and kidney disease in a temperate city.^4^^,^^33^ In addition, there are reports that high ambient temperature is associated with kidney stone occurrence in some populations.^26^^,^^34^^,^^35^

Our finding of an association between occupational heat stress and kidney disease is new for Thailand. However, the potential links of high occupational heat exposure to health effects in Thailand was recently discussed in an article by Langkulsen et al.^17^ The heat stress effects detected in our study translate to a significant public health burden of preventable heat-related kidney disease among Thai workers. This is particularly important at present because we expect that the existing problem will worsen if global warming continues and that workplaces will become even more thermally stressful. More attention is needed to the health of tropical workers, especially older male workers doing physical jobs during hot weather or under prolonged heat stress. Maintaining hydration must be emphasized for physical workers in Thailand, and this requires a health-behavior intervention. In addition, construction of buildings for workplaces must account for indoor climate while minimizing the use of energy-consuming air conditioning systems.

In our longitudinal analysis of Thai Cohort Study data, we conclude there is an association between exposure to occupational heat stress and kidney disease among men. The underlying mechanisms require further study. A rapid increase is kidney disease is already occurring in tropical Thailand. Heat stress should be regarded as a potential cause, and global warming will increase the risk.
